# Distinct microbial signatures and their predictive value in recurrent acute pancreatitis: insights from 5-region 16S rRNA gene sequencing

**DOI:** 10.3389/fimmu.2025.1558983

**Published:** 2025-02-28

**Authors:** Qi-Wen Wang, Haorui Zheng, Yang Yang, Xinyao Chang, Zengkan Du, Zi-ning Hang, Zhao-Shen Li, Zhuan Liao

**Affiliations:** Department of Gastroenterology, Shanghai Institute of Pancreatic Diseases, Changhai Hospital, Naval Medical University, Shanghai, China

**Keywords:** acute pancreatitis, recurrent acute pancreatitis, microbiome, 16S rRNA gene sequencing, *Staphylococcus hominis*

## Abstract

**Background:**

Recurrent acute pancreatitis (RAP) poses significant clinical challenges, with 32.3% developing to chronic pancreatitis within 5 years. The underlying microbial factors contributing to RAP remain poorly understood. This study aims to identify blood microbial signatures associated with RAP and explore the potential microbial predictors for RAP.

**Methods:**

In this prospective cohort, 90 acute pancreatitis patients are classified into non-recurrent acute pancreatitis (NRAP, n=68) and RAP (n=22) groups based on the number of pancreatitis episodes. Microbial composition of blood samples is analyzed using 5-region (5R) 16S rRNA gene sequencing. Key microbial taxa and functional predictions are made. A random forest model is used to assess the predictive value of microbial features for RAP. The impact of *Staphylococcus hominis (S. hominis)* on RAP is further evaluated in an experimental mouse model.

**Results:**

Linear discriminant analysis effect size (LEfSe) analysis highlights significant microbial differences, with *Paracoccus aminovorans*, *Corynebacterium glucuronolyticum* and *S. hominis* being prominent in RAP. Functional predictions indicate enrichment of metabolic pathways in the RAP group. Random forest analysis identifies key microbial taxa with an AUC value of 0.759 for predicting RAP. Experimental validation shows that *S. hominis* exacerbates pancreatic inflammation in mice.

**Conclusions:**

This study identifies distinct clinical and microbial features associated with RAP, emphasizing the role of specific bacterial taxa in pancreatitis recurrence. The findings suggest that microbial profiling could enhance the diagnosis and management of RAP, paving the way for personalized therapeutic approaches.

## Introduction

Acute pancreatitis (AP) is an acute inflammation of the pancreas caused by etiologic factors such as gallstones, alcohol use, and hypertriglyceridemia that can lead to significant morbidity and mortality (1). The incidence of AP is estimated at 13 to 49 cases per 100,000 persons per year (2). Despite advances in medical care, a substantial proportion of AP patients experience recurrent episodes, termed recurrent acute pancreatitis (RAP), with a reported recurrence rate of 11%–36% (3, 4). RAP represents a critical turning point in AP-RAP-chronic pancreatitis (CP) continuum, as it remarkably increases the risk of life-threatening complications, up to 32.3% progression to CP within 5 years and substantial socioeconomic burdens, underscoring an urgent need for novel predictive biomarkers and precision intervention paradigms (5–10).

Recent decades have witnessed the gut-pancreas axis dysbiosis in pancreatitis pathogenesis. AP patients exhibit decreased commensal *Lactobacillus* and increased pathogenic *Enterobacteriaceae*, correlating with systemic inflammation and pancreatic necrosis (11, 12). Crucially, researches have reported that there is an “authentic circulating microbiome”—the collection of microbial DNA, RNA, and other microbial components found in the bloodstream (13–15). Païssé et al. confirmed that there was a stable microbial community with low biomass (< 0.1pg/μL) but taxonomically diverse in the blood of healthy people, mainly from *Proteobacteria* phylum (80%) and *Actinobacteria* phylum (10%) (16). Unlike the gut microbiome, blood microbial communities may originate from intestinal translocation, or infection or bacteremia, acting as “stealth contributors” to inflammation (17–20). However, conventional single-region 16S sequencing (e.g., V3-V4) or shotgun metagenomics lack resolution to detect low-biomass blood microbiota as high abundance of eukaryotic DNA products complicate dramatically the sequencing of bacterial DNA. Besides, no studies have explored blood microbiome dynamics between AP and RAP; and the predictive capacity of blood microbial signatures for RAP remains uncharacterized.

In light of these challenges, we collect serum samples from patients within 24 hours of the onset of AP, and divide patients into NRAP and RAP groups based on medical history. By pioneering 5-region (5R) 16S rDNA sequencing—a high-resolution approach especially for the detection of microbial samples with low biomass (21), we aim to decipher blood microbiome dynamics across AP-RAP trajectories, identify RAP-specific microbial signatures via 5R-enhanced resolution, and develop machine learning models integrating microbial biomarkers with clinical variables for RAP prediction, and investigate the effects of specific pathogenic bacteria on RAP in animal experiments.

## Methods

### Human sample collection

This is a single-center, cross-sectional study. Patients are enrolled in the First Affiliated Hospital of Naval Medical University, Shanghai, China, between September 2022 and April 2023. Serum samples are collected from patients diagnosed with AP according to the diagnostic criteria within 24 hours of disease onset (1). All participants provide informed consent. According to the diagnostic criteria for RAP, which includes clinical symptoms, serological, and imaging tests confirming AP, with ≥ 2 episodes, no permanent histological changes such as exocrine or endocrine dysfunction, fibrosis, or calcification, and an interval of ≥ 3 months between the two AP episodes, AP patients are further classified into NRAP and RAP groups (9). The major exclusion criteria are chronic pancreatitis, inflammatory bowel disease, immunosuppressive disease, cancer, the use of antibiotics within two months of enrollment. Detailed demographic and clinical information, including age, gender, medical history, and laboratory findings, are recorded for each patient. Ethical approval is obtained from the Shanghai Changhai Hospital Ethics Committee (CHEC2021-198). The samples are stored at -80°C refrigerator before analysis.

### DNA extraction and 5R 16S rRNA gene sequencing

DNA is extracted from serum samples using standardized protocols to ensure high-quality genetic material. 5R 16S rRNA sequencing is used in the study to mitigate host DNA interference and facilitate microbiota detection in samples with low microbial content but high host proportions. The experiment utilizes polymerase chain reaction (PCR) to amplify variable regions of the 16S rRNA gene (V2, V3, V5, V6, and V8). The purified PCR products are evaluated using an Agilent 2100 Bioanalyzer (Agilent Technologies, USA) and the Illumina 16S Metagenomic Sequencing Library (Kapa Biosciences, Woburn, MA, USA), ensuring that the qualified library concentration was above 0.3 ng/μL. Paired-end sequencing is performed using the Illumina NovaSeq 6000 sequencing platform in PE150 sequencing mode, generating 20 Gb/sample.

### Bioinformatics analysis and visualization

The sequencing data is analyzed using the Short MUltiple Regions Framework (SMURF) analysis pipeline, employing the optimized Greengenes (May 2013 version, Second Genome) database. The purified amplicon sequence variants (ASVs) are generated to identify microbial profiles. Alpha diversity analysis is conducted based on the resulting species-level abundance tables, using indices such as observed_species, Shannon, Simpson, Chao1, and goods_coverage to evaluate intra-sample diversity. Beta diversity is assessed by calculating two distances (Bray-Curtis) and performing six analyses to evaluate inter-sample/group diversity. A Venn diagram showing overlapping ASVs between the two groups is created using the VennDiagram package in R (version 4.3.1). The ggplot2 package is used for visualization. Linear discriminant analysis effect size (LEfSe) is used to compare microbial compositions, with thresholds set at an LDA score >3 and *P* < 0.05.

### Microbiota function prediction and clinical correlation analysis

The functional potential of the microbiota is predicted using PICRUSt2 software (Version 2.4.1). The predicted functional profiles are analyzed for differences using STatistical Analysis of Metagenomic Profiles (STAMP) (version 2.1.3, Beiko Lab). Correlation analysis between clinical information and microbiota is performed using Spearman’s rank correlation coefficient and then visualized using the pheatmap package (Version 1.0.12, CRAN) in R (version 4.3.1), which assesses the strength and direction of association between clinical variables and microbial abundance.

### Disease diagnostic model construction and validation

Based on the sequencing data, a random forest regression model for RAP is constructed in R (version 4.3.1) from the randomForest package, with 70% of the samples (n=63) assigned to the training cohort and the remaining 30% (n=27) to the validation cohort. Ten-fold cross-validation is applied to the training cohort. The most important variables are used to build the predictive model, and ROC curves are calculated to distinguish between NRAP and RAP patients. The confidence intervals for the ROC curves are calculated using the pROC package in R (version 4.3.1).

### Animal experiment

Male C57BL/6 mice (6 - 8 weeks old) obtained from GemPharmatech Co., Ltd., (Nanjing, China), are treated with broad-spectrum antibiotics (ampicillin 1 g/l, neomycin 1 g/l, metronidazole 1 g/l, and vancomycin 0.5 g/l) in their drinking water for 4 weeks before experiment to deplete gut and systemic microbiota, as previously described (22). After antibiotic treatment, mice are randomly divided into PBS-gavaged group (n=8) and *Staphylococcus hominis* (*S. hominis*) - gavaged group (n=8). Mice are administered 200 ul PBS/mice only or at a dose of 10^7^ colony-forming units (CFU) of *S. hominis* intragastrically once every two days for 2 weeks. This colonization protocols ensures dominance of *S. hominis* over potential residual microbes (23). After the first attack of AP with 8 hourly injections of 100 ug/kg caerulein, mice are allowed to recover for 7 days and receive the second attack to induce RAP (24). At 24 h after the first injection of caerulein in second period, mice are sacrificed and analyzed. All animal care and experimental protocols are approved by the Animal Ethics Committee of Shanghai Changhai Hospital Ethics Committee (CHEC (A.E) 2022-008).

### Enzyme-linked immunosorbent assays

Mice serum is obtained by centrifuging whole blood samples. Serum levels of Amylase (Abcam, ab102523), IL-1β (Abcam, ab197742) and TNF-α (Abcam, ab102523) is measured using commercial ELISA kits according to manufacturer’s instructions.

### Histological analysis

Formaldehyde-fixed pancreas is embedded in paraffin, sectioned into slices, and stained with hematoxylin-eosin (HE). Pancreatic histological score is assessed under a light microscope (Olympus, Tokyo, Japan) according to Rongione’s standard (25). Inflammatory cells in pancreas are evaluated by detection of F4/80 by immunohistochemistry. Briefly, slides are heated in a 70°C oven for 30 min and then deparaffinized in xylene, followed by rehydration through a graded ethanol series. After antigen repairing procedure, slides are incubated with F4/80 antibody (Abcam, ab300421, 1:500) at 4 °C overnight. After washing with PBS, slides are developed with DAB substrate and counterstained with hematoxylin. The IHC positive staining is analyzed by ImageJ (Version 1.8, NIH, USA).

### Flow cytometry

Pancreatic tissue samples are harvested and digested in collagenase IV solution at 37°C for 30 min. Pancreatic cells are subsequently filtered through a 70 μm cell strainer. After washing and lysis of erythrocytes, Single-cell suspensions are incubated for 15 min at room temperature in stain buffer with the following antibodies for surface markers: mCD45 (#103108, BioLegend, FITC conjugate), mCD11b (#101230, Biolegend, PerCP conjugate) and mF4/80 (#123116, BioLegend, APC conjugate). Sample acquisition is carried out on Cytoflex flow cytometer (Beckman Cytoflex S, USA) and data are analyzed using FlowJo 10.8 (BD Biosciences, San Jose, CA).

### Statistical analysis

Clinical characteristics of the two patient groups are analyzed using SPSS software (version 19.0, IBM Corp, USA). For normally distributed continuous variables, data are presented as mean ± standard deviation (SD); for non-normally distributed continuous variables, data are presented as median (interquartile range [IQR]). Categorical variables are presented as numbers (percentages). The *P*-value for categorical variables is calculated using the chi-square test or Fisher’s exact test. Continuous variables are analyzed using the t-test or the non-parametric Kruskal-Wallis test. A two-sided *P*-value of less than 0.05 is considered statistically significant.

## Results

### Clinical baseline information

90 patients are included in this study, classified into 68 NRAP cases and 22 RAP cases based on recurrence history. The demographic and clinical characteristics of the participants are summarized in **Table 1**. No significant differences are found among the key clinical variables, including age, gender, and etiology. The NRAP group shows higher levels of LDH compared to the RAP group (median 311 U/L, IQR [198–492] vs. median 219 U/L, IQR [155–281]; *P* = 0.021). Similarly, BUN levels are higher in the NRAP group than in the RAP group (median 5.1 mmol/L, IQR [4.0–6.6] vs. median 4.5 mmol/L, IQR [3.2–4.9]; *P* = 0.025). The proportion of severe acute pancreatitis patients is higher in the NRAP group compared to the RAP group (35.3% vs. 22.7%; *P* = 0.138), but this difference is not statistically significant. The duration of hospitalization shows no significant difference between the two groups (NRAP: median 10 days, IQR [5-21] vs. RAP: median 8 days, IQR [5-12]; *P* = 0.171).

**Table 1:** Demographic and clinical characteristics of NRAP and RAP patients.

Factors	NRAP (n=68)	RAP (n=22)	*P*
Age (years), median (IQR)	48 (36 - 61)	51 (39 - 58)	0.735
Male gender, n (%)	38 (56)	15 (68)	0.311
Laboratory findings			
Triglyceride, median (IQR), mmol/L	1.26 (0.64 - 3.81)	1.58 (0.81 - 3.99)	0.866
CRP, median (IQR), mg/L	209.0 (73.7 - 305.5)	170.0 (83.8 - 204.5)	0.381
Blood Amylase, median (IQR), U/L	503 (237 - 1047)	188 (81 - 365)	0.055
Blood Lipase, median (IQR), U/L	278.7 (153.9 - 409.5)	111.4 (72.8 - 286.7)	0.254
LDH, median (IQR), U/L	311 (198 -492)	219 (155 - 281)	**0.021**
BUN, median (IQR), mmol/L	5.1 (4.0 - 6.6)	4.5 (3.2 - 4.9)	**0.025**
ALT, median (IQR), U/L	26 (15 - 75)	19 (15 -30)	0.078
Scr, median (IQR), umol/L	64.5 (56.0 - 88.0)	62.0 (50.0 - 74.0)	0.270
Hospital stay (days), median (IQR)	10 (5-21)	8 (5-12)	0.171
Disease severity, n (%)			
MAP	21 (30.9)	11 (50.0)	0.105
MSAP	23 (33.8)	6 (27.3)	0.570
SAP	24 (35.3)	5 (22.7)	0.276
Etiology, n (%)			
Biliary	31 (45.6)	6 (27.3)	0.131
Hypertriglyceridemia	17 (25.0)	9 (40.9)	0.155
Alcohol consumption	4 (5.9)	1 (4.5)	0.813
Other	16 (23.5)	6 (27.3)	0.724

TG, Triglyceride; CRP, C-reactive protein; LDH, lactate dehydrogenase; BUN, Blood Urea Nitrogen; ALT, alanine aminotransferase; Scr, serum creatinine; MAP, mild acute pancreatitis; MSAP, moderately severe acute pancreatitis; SAP, severe acute pancreatitis. Bold values indicate statistical significance at *P*<0.05.

### Microbial profile of NRAP and RAP patients

**Figure 1:**
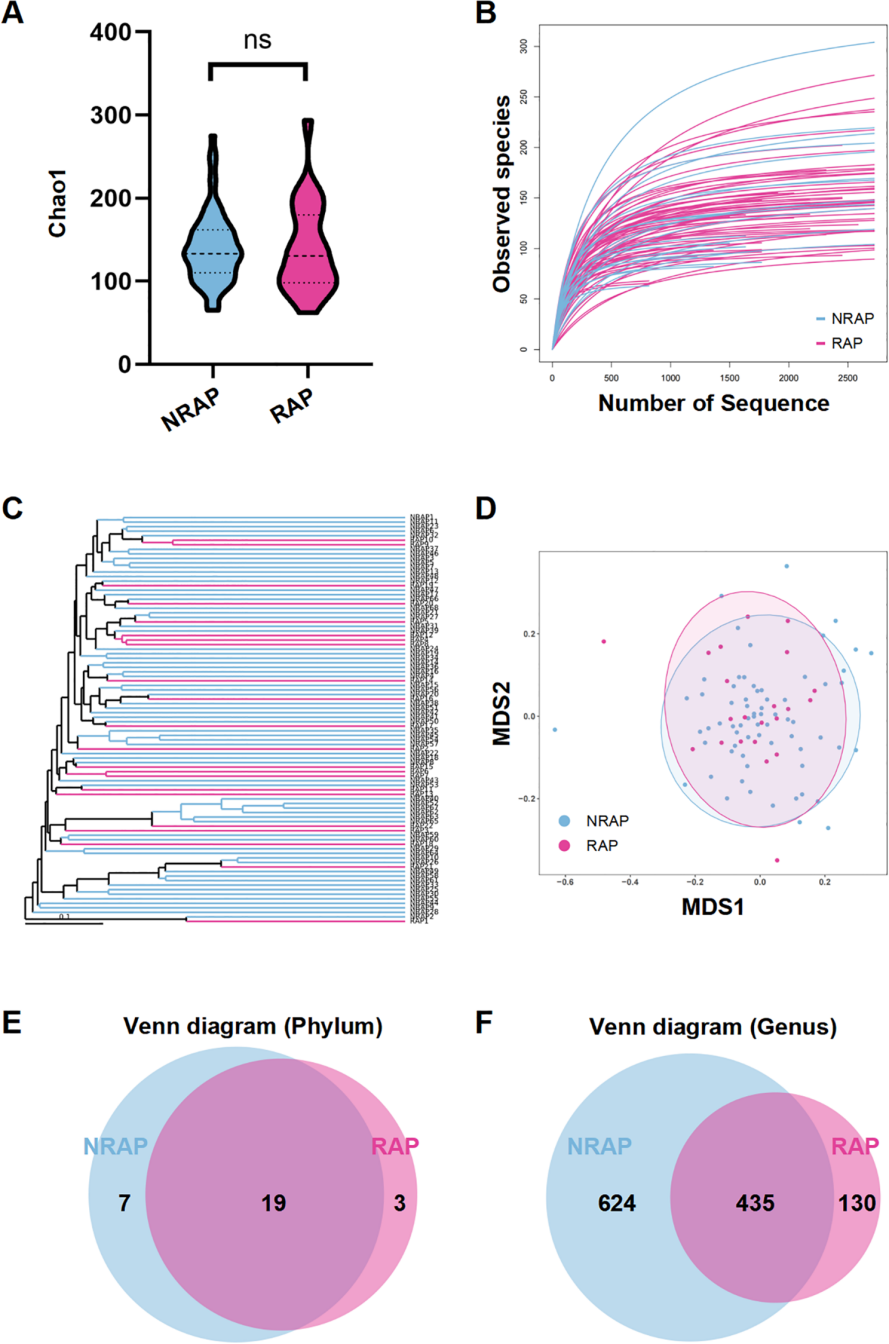
Comparison of microbial diversity between NRAP and RAP patients. **(A)** Alpha-diversity analysis; **(B)** Species richness rarefaction curve; **(C)** NMDS plot; **(D)** UPGMA clustering analysis; Venn diagram of shared and unique bacterial phyla **(E)** and genera **(F)** in NRAP and RAP groups. Top bars: ns, *P* > 0.05.

The microbial compositions of serum samples from NRAP and RAP patients are characterized by 5R 16S rRNA gene sequencing. The sequencing quality is eligible, with no samples discarded and average reads count of 33,312 per sample. As shown in **Figure 1A**, there is no significant difference in α-diversity between the two groups, indicating that the overall composition of blood microbiota is similar. The species richness rarefaction curve gradually levels off, indicating a reasonable number of individual samples (**Figure 1B**). NMDS and UPGMA cluster analysis based on the UniFrac algorithm shows no significant difference in microbial β-diversity between the two groups (**Figures 1C, D**). α/β diversity indices reflect the unique nature of blood microbiota, which are inherently similar in dominant phyla. Venn diagrams show the common and unique phyla and genera detected in the NRAP and RAP groups (**Figures 1E, F**).

**Figure 2:**
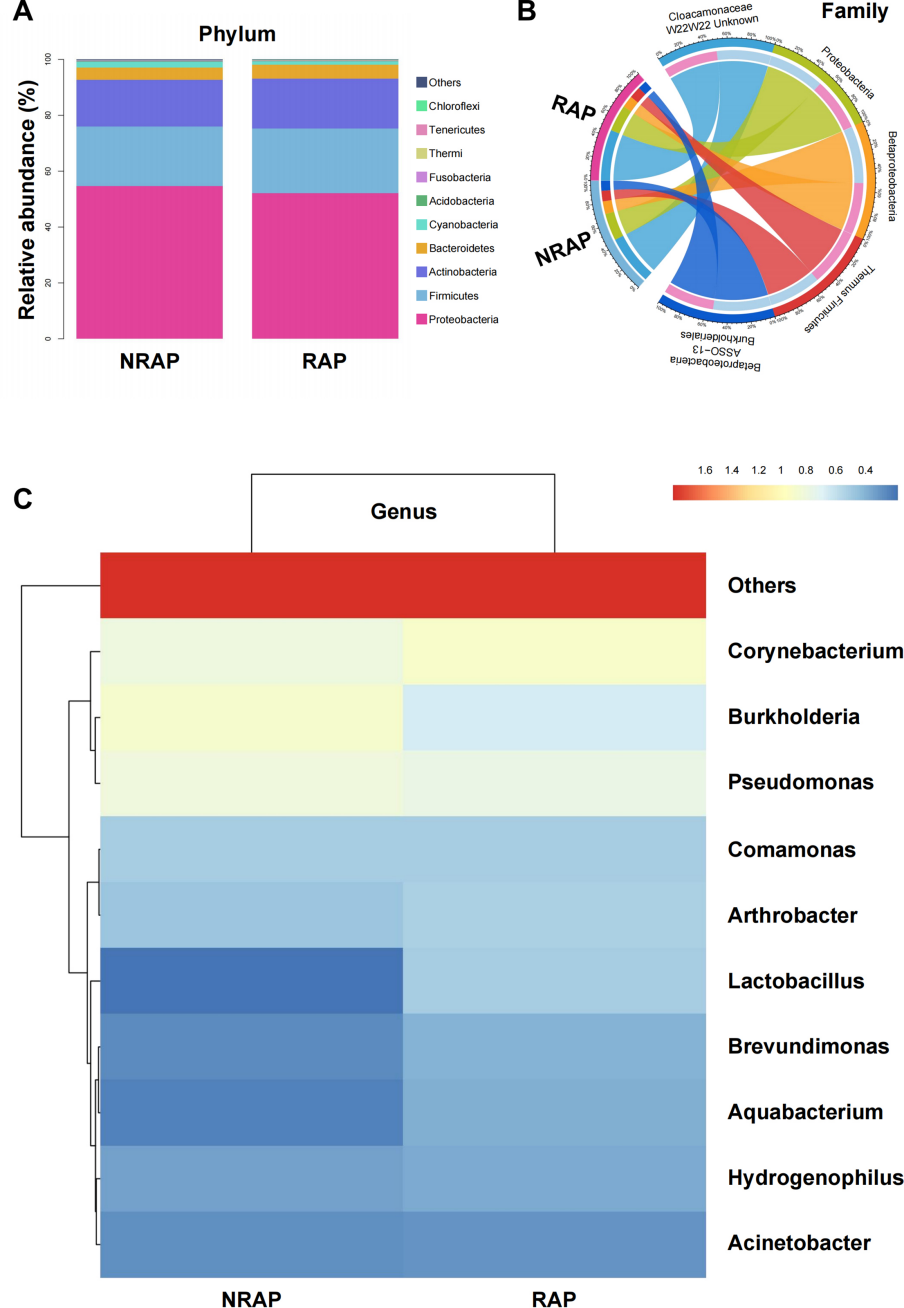
Relative Abundance of Major Bacterial Taxa in NRAP and RAP patients. **(A)** Stacked bar chart of top 10 bacterial phyla in NRAP and RAP groups; **(B)** Chord diagram showing associations of microbial families between NRAP and RAP groups; **(C)** Top 10 genera profiles comparing NRAP and RAP groups.

**Figure 2A** presents a stacked bar chart of the top 10 relative abundance bacterial species at the phylum level in NRAP and RAP groups, mainly composed of *Proteobacteria* (NRAP: 54.74% vs. RAP: 52.09%), *Firmicutes* (NRAP: 21.28% vs. RAP: 23.24%), and *Actinobacteria* (NRAP: 16.65% vs. RAP: 17.80%). Minor phyla (e.g., *Bacteroidetes* and *Cyanobacteria*) collectively account for < 8% of the microbiota. A chord diagram at the family level visualizes the microbial community composition and the associations between the two groups (**Figure 2B**). The top 10 genera profiles are shown in **Figure 2C**. *Burkholderia* is more frequent in the NRAP group than in the RAP group (NRAP: 8.67% vs. RAP: 5.25%), while the relative abundance of *Corynebacterium* is higher in the RAP group (NRAP: 7.11% vs. RAP: 8.95%). NRAP group shows a lower relative abundance of *Lactobacillus* when compared with the RAP group (NRAP: 1.69% vs. RAP: 3.64%).

### Taxonomic characteristics of NRAP and RAP patients

**Figure 3:**
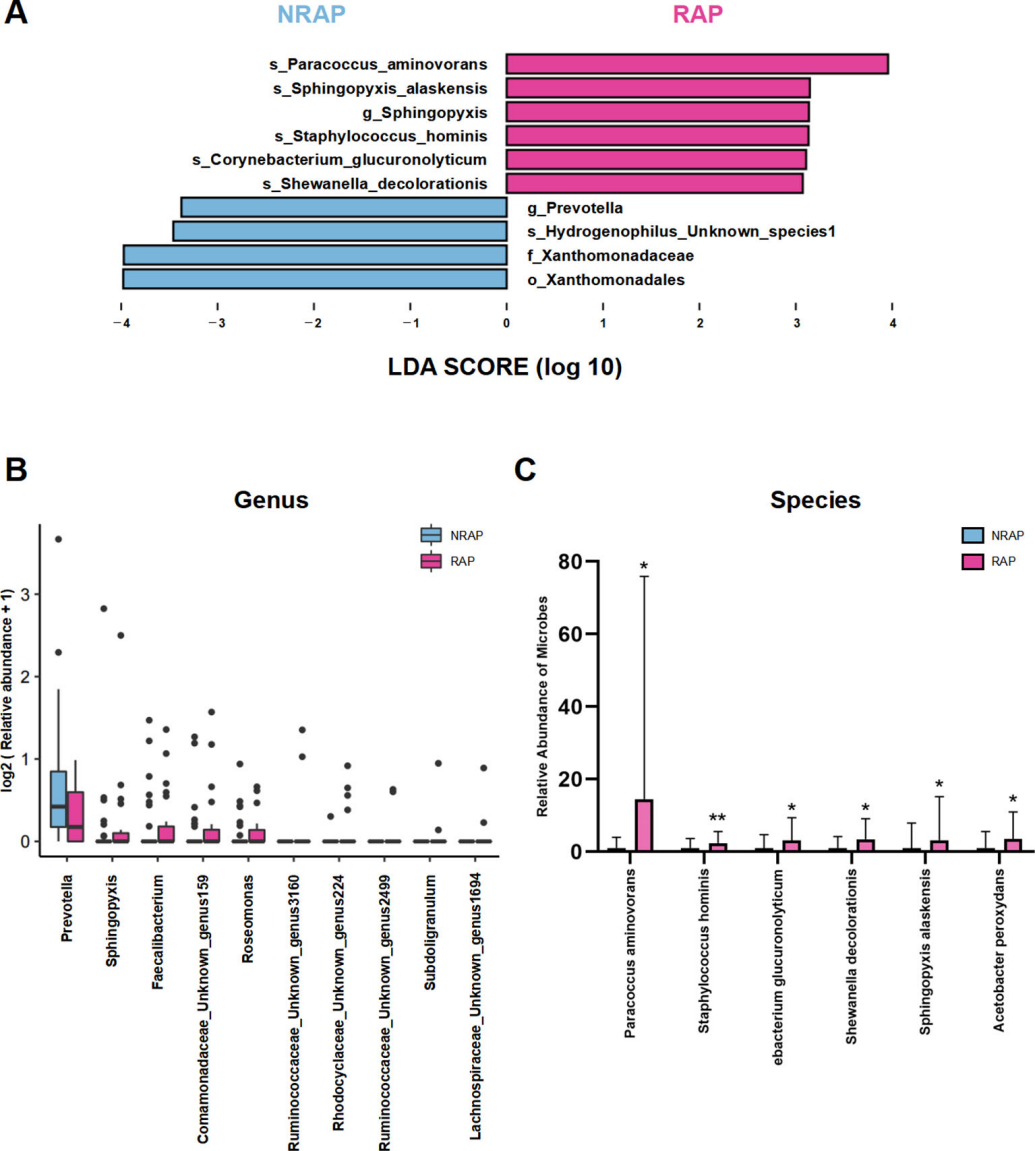
Differentially abundant taxa between NRAP and RAP patients. **(A)** LEfSe analysis; **(B)** Boxplot of top 10 genera with significant differences between NRAP and RAP groups; **(C)** Comparison of specific bacterial species abundances in NRAP and RAP groups. Top bars: *, wilcox-test *P* value < 0.05; **, wilcox-test *P* value < 0.01.

LEfSe analysis depicts significant differences in bacterial taxa between the NRAP and RAP groups (**Figure 3A**). The key microbial taxa in the NRAP group include *g_Prevotella*, *f_Xanthomonadaceae*, and *o_Xanthomonadales*, whereas in the RAP group, *g_Sphingopyxis* is the dominant taxon. A boxplot visually displays the top 10 ranking genera with significant differences (**Figure 3B**). At the species level, compared to the NRAP group, the RAP group shows higher abundances of *Paracoccus aminovorans* (2.172% vs. 0.150%, *q* = 0.048), *Staphylococcus hominis* (0.503% vs. 0.210%, *q* = 0.01), and *Corynebacterium glucuronolyticum* (0.436% vs. 0.140%, *q* = 0.03) (**Figure 3C**).

### Microbial functions and correlation with clinical indicators in NRAP and RAP patients

**Figure 4:**
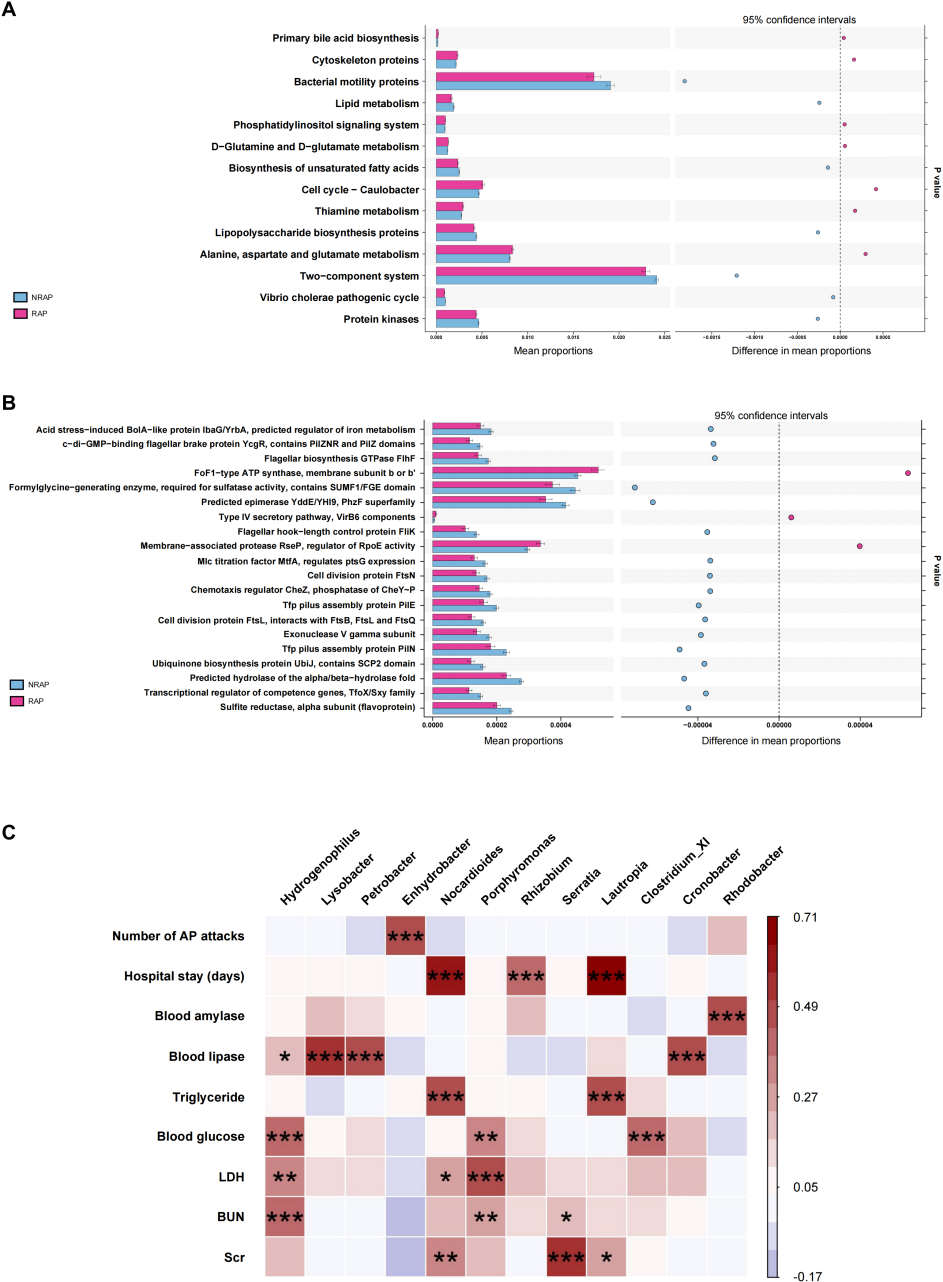
Functional predictions and clinical correlations of blood microbiota in NRAP and RAP patients. **(A)** KEGG pathway enrichment; **(B)** COG pathway enrichment; **(C)** Spearman correlation heatmap showing associations between microbiota and clinical indicators. Top bars: *, *P* value < 0.05; **, *P* value < 0.01, ***, *P* < 0.001.

Using PICRUSt analysis to predict gene functions of the microbiota, we find that the differential microbial community in the RAP group is enriched in KEGG pathways such as alanine, aspartate and glutamate metabolism, thiamine metabolism, and cell cycle-caulobacter; and COG pathways such as FoF1−type ATP synthase, membrane subunit b or b’ when compared to the NRAP group (**Figures 4A, B**).

Spearman correlation analysis is conducted to identify associations between blood microbiota and clinical indicators, including demographic characteristics, laboratory tests, duration of hospitalization, and number of pancreatitis episodes (**Figure 4C**). At the genus level, *Enhydrobacter* is positively correlated with the number of AP attacks (r = 0.45, *P* < 0.001). *Lautropia* shows a strong positive correlation with hospitalization duration (r = 0.71, *P* < 0.001) and a moderate positive correlation with triglyceride (TG) (r = 0.48, *P* < 0.001). Additionally, *Nocardioides* is moderately positively correlated with both hospitalization duration and TG, with statistical significance (r = 0.58, *P* < 0.001; r = 0.47, *P* < 0.001).

### Potential microbial features predictive of RAP

**Figure 5:**
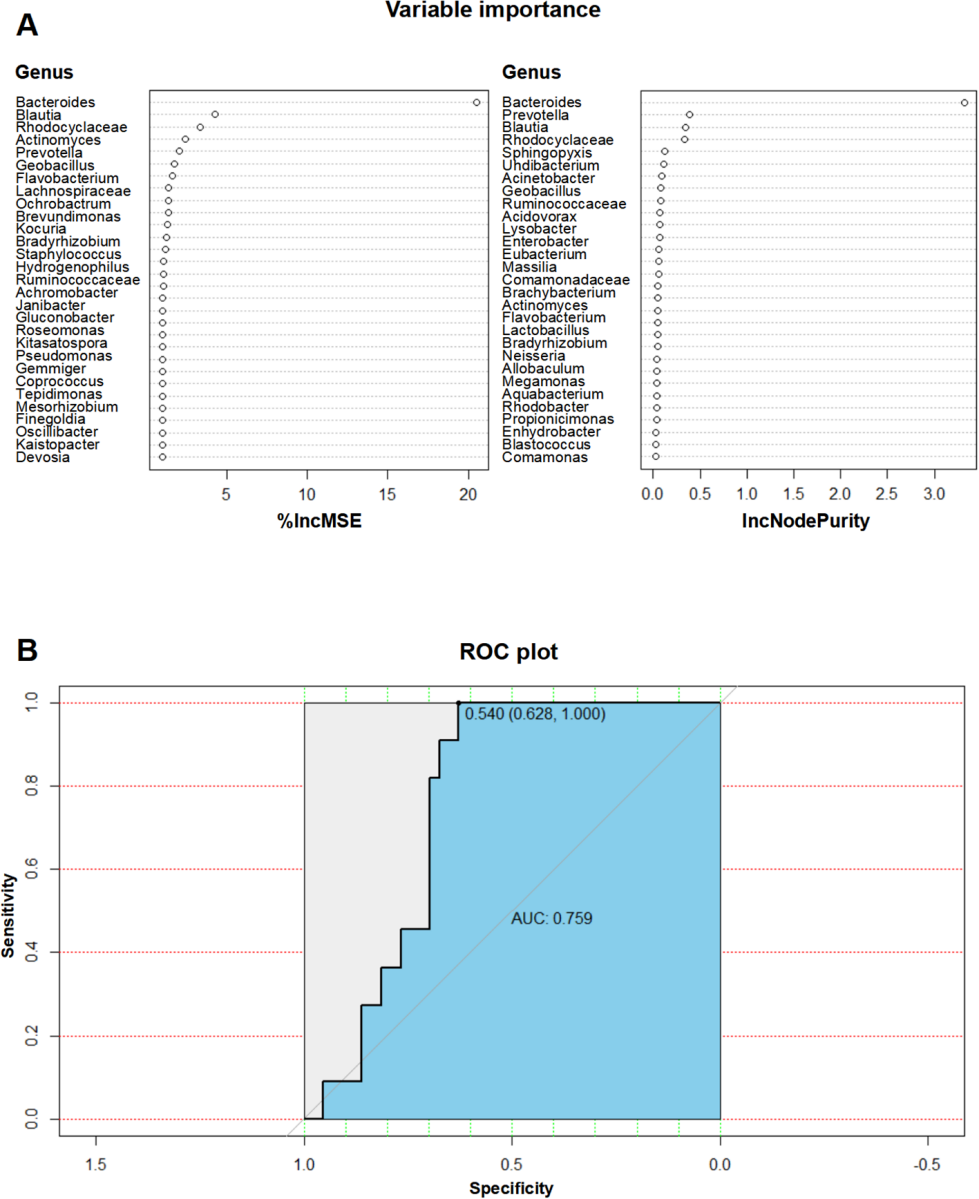
Predictive value of microbial features for RAP diagnosis. **(A)** IncMSE and IncNodePurity analysis from the random forest model identifying key taxa for RAP prediction; **(B)** ROC curve of the diagnostic model with an AUC value of 0.759 for RAP prediction.

To investigate the value of blood microbial features in predicting RAP, we use a random forest regression model to identify taxa associated with RAP. Increase in Mean Squared Error (%IncMSE) identifies *Bacteroides* (4.3%), *Blautia* (3.3%), and *Rhodocyclaceae* (2.4%) as top predictors (**Figure 5A**). Increase in Node Purity (IncNodePurity) reveals that *Bacteroides* (0.38), *Prevotella* (0.35), and *Blautia* (0.33) show strong classification power (**Figure 5A**). In the validation cohort, the diagnostic model has an AUC value of 0.759 (95% CI: 0.6346 - 0.8834, cutoff > 0.540, sensitivity: 100%, specificity: 62.8%), demonstrating moderate discriminative power (**Figure 5B**).

### *S. hominis* exacerbates experimental RAP in mice

**Figure 6:**
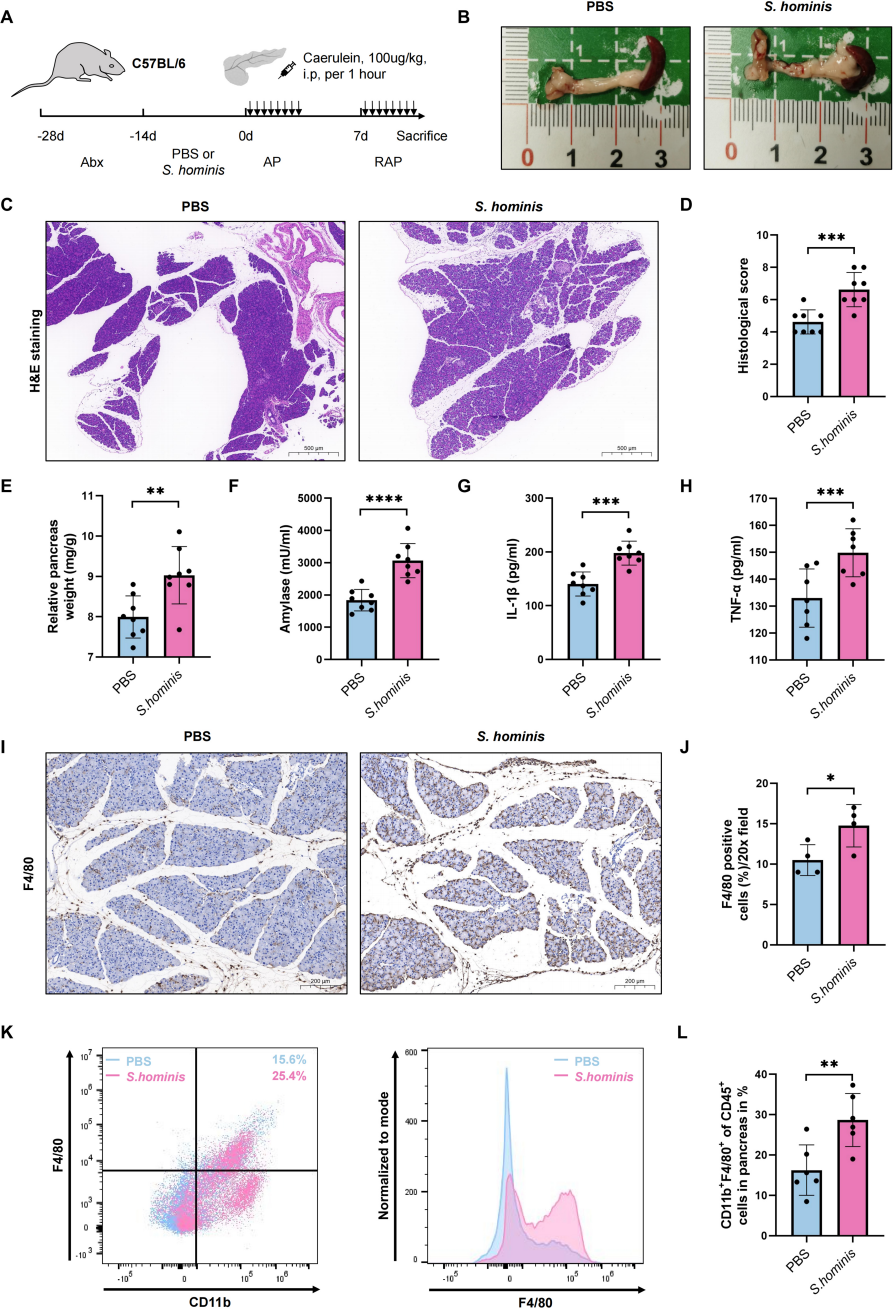
Impact of S. hominis on pancreatitis severity in a mouse RAP model. **(A)** Experimental design for PBS or S. hominis-gavaged mice in the RAP model; **(B)** Gross morphological appearance of pancreatitis; **(C, D)** H&E staining and histological scores of pancreatic tissues; **(E)** Relative pancreatic weight ratio; **(F–H)** Serum levels of amylase, TNF-α, and IL-1β; **(I, J)** Immunohistochemical detection of macrophage infiltration in the pancreas; **(K, L)** Quantification of CD11b+F4/80+cell infiltration in the pancreas via flow cytometry. Top bars: *, *P* < 0.05; **, *P* < 0.01; ***, *P* < 0.001; ****, *P* < 0.0001.

To further validate the hypothesis that microbes associated with RAP can participate in disease progression, *S. hominis*, a Gram-positive bacterium from the *Staphylococcus* genus, is selected in this study based on the LEfSe analysis (**Figure 3A**), the top five differential species data between the two groups (**Figure 3C**), and previous literature reports. Antibiotic-treated, microbiota-depleted mice are gavaged with *S. hominis* or PBS as control for two weeks, and then induce with caerulein to create an RAP model (**Figure 6A**). The relative pancreatic weight ratio in the *S. hominis* group is higher than in the PBS group, indicating more severe pancreatic inflammation (**Figures 6B, E**). Histological evaluation of the pancreas shows more edema, significant inflammatory cell infiltration, and higher pancreatic tissue scores in the *S. hominis* group compared to the PBS group (**Figures 6C, D**). Serum biochemical analysis reveals elevated levels of serum amylase, pro-inflammatory cytokines TNF-α and IL-1β in the *S. hominis* group compared to the PBS group (**Figures 6F–H**). Immunohistochemical detection using F4/80 identifies increased macrophage infiltration in the pancreas of the *S. hominis* group compared to the PBS group (**Figures 6I, J**). Flow cytometry further confirms increased macrophage infiltration in the pancreas of the *S. hominis* group relative to the PBS group (**Figures 6K, L**).

## Discussion

While the gut-pancreas axis has been extensively studied in pancreatitis pathogenesis—particularly focusing on gut dysbiosis-driven systemic inflammation, bacterial translocation, and entero-pancreatic metabolite crosstalk (26–28)—our work uncovers a previously unrecognized dimension of microbial influence: the blood microbiome as an independent mediator of RAP progression. Additionally, our findings suggest that real-time blood microbiome monitoring may offer clinical potential in acute care settings, underscoring its broader translational relevance compared to stool-based methods.

The clinical characteristics of the NRAP and RAP groups reveal subtle differences among microbial overall diversity, richness, and community structure. While the similar dominant phylum in blood microbiota, the nature of low-biomass rare taxa and technical ceiling may dilute subtle pathogen-specific shifts, LEfSe and functional analyses reveal that low-abundance taxa act in RAP through specific virulence and metabolic reprogramming.

The RAP group exhibited significant enrichment of *Corynebacterium glucuronolyticum*. *Corynebacterium* species, club-shaped gram-positive rods, are commonly found in animal hosts and are part of healthy human skin flora. Recently, they have recently been identified as causative agents of severe bloodstream infections, infective endocarditis, pneumonia and meningitis (29–32). Furthermore, growing evidence suggests that *Corynebacterium* species act as opportunistic pathogens in long-term hospitalized patients and have developed to drug resistance (33, 34). Its predominance in RAP suggests that they may be high-risk pathogens. *Pseudomonas aeruginosa*, identified by LEfSe as a top differential species, is well-known for causing acute or chronic infections in immunocompromised individuals and poses significant treatment challenges due to its rapid acquisition of drug resistance.

The enrichment of alanine, aspartate, and glutamate metabolis in the RAP group suggests a microbial role in modulating amino acid homeostasis, which is critical for pancreatic acinar cell survival and inflammation regulation. Elevated glutamate levels, for instance, are known to activate *N*-methyl-D-aspartate (NMDA) receptors in pancreatic neurons, exacerbating neurogenic inflammation and pain perception in chronic pancreatitis (35, 36). Similarly, the prominence of thiamine metabolism implies microbial involvement in energy production pathways, potentially compensating for oxidative stress-induced mitochondrial dysfunction in recurrent injury (37). Notably, the enrichment of FoF1-type ATP synthase highlights microbial adaptations to nutrient-depleted environments, possibly facilitating bacterial persistence in systemic circulation during recurrent attacks (38, 39). The positive correlation between *Enhydrobacter* abundance and AP attack frequency (r = 0.45, *P* < 0.001), which aligns with Szabó et al. observing *Enhydrobacter* abundance in septic patients (40). *Lautropia*’s strong association with prolonged hospitalization (r = 0.71) suggests its potential to cause invasive disease, which accords with another study obtaining five clinical strains of *Lautropia* from normally sterile sites (blood, peritoneal fluid) (41). Our Random Forest model achieves an AUC value of 0.759 with high sensitivity for predicting RAP. This underscores the potential for blood microbial profiling in clinical settings to predict and manage RAP effectively.

*Staphylococcus hominis*, another dominant species in the RAP group, represents the second most prevalent Coagulase-Negative *Staphylococcus* species (CoNS) colonizing human skin microbiota (42). However, it has been reported to be an opportunistic pathogen implicated in bloodstream infections, endocarditis and peritonitis (43, 44). Although the precise mechanisms by which *S. hominis* exacerbates RAP in our research remain to be fully elucidated, we hypothesize multiple synergistic mechanisms involved. First, its ability to form biofilms could facilitate colonization in necrotic pancreatic foci or pancreatic ducts (45). Biofilm-associated persistence might shield the bacterium from immune clearance and antibiotic penetration, thereby sustaining localized inflammation and tissue damage. Second, *S. hominis*-derived enzymes, including proteases and lipases (46), could directly degrade pancreatic parenchymal cells or amplify trypsinogen activation, potentially accelerating autodigestion and necrosis. Third, metabolites produced by *S. homini* could modulate macrophage polarization, skewing the immune response toward a hyperinflammatory state. Finally, in the context of pancreatitis-associated gut barrier dysfunction (47), *S. hominis* overgrowth in the gut-pancreas axis might disrupt microbial homeostasis, promoting bacterial translocation and secondary infection.

While our study provides valuable insights, there are several limitations to consider. First, the sample size was relatively small, which may limit the generalizability of our findings. Larger cohort studies are needed to validate these results. Second, a critical consideration in microbiome studies is the potential influence of pharmacological interventions. In our cohort, though the enrollment of patients accomplishes within 24 hours of disease onset, and patients within two months antibiotics use are excluded, medications may still confound our findings. Besides, our study design is cross-sectional, making it challenging to establish causality between the identified microbial signatures and RAP. Longitudinal studies would be beneficial to assess the temporal relationship between microbial changes and pancreatitis recurrence. In addition, while we identify exacerbation of RAP in *S. hominis*-infected mice, the underlying mechanisms by which this bacterium influences pancreatitis remain unclear. Future studies should focus on elucidating these mechanisms through in-depth functional analyses and experimental models.

In conclusion, our study first elucidates distinct circulating bacterial taxa in RAP, uncovering bacteria taxa which may drive disease recurrence, and laying the groundwork for developing microbiota-directed diagnostics and targeted therapies to disrupt the AP-RAP-CP continuum.

## Data Availability

The datasets presented in this study have been deposited in the NCBI Sequence Read Archive (SRA) under the BioProject accession number PRJNA1224315. These data will be accessible starting from the publication date via the following link:https://www.ncbi.nlm.nih.gov/sra/PRJNA1224315.
